# The Dual Nature of Type I and Type II Interferons

**DOI:** 10.3389/fimmu.2018.02061

**Published:** 2018-09-11

**Authors:** Amanda J. Lee, Ali A. Ashkar

**Affiliations:** Department of Pathology and Molecular Medicine, McMaster Immunology Research Centre, McMaster University, Hamilton, ON, Canada

**Keywords:** type I interferon, interferon-γ, innate immunity, virus infection, immunoregulation

## Abstract

Type I and type II interferons (IFN) are central to both combating virus infection and modulating the antiviral immune response. Indeed, an absence of either the receptor for type I IFNs or IFN-y have resulted in increased susceptibility to virus infection, including increased virus replication and reduced survival. However, an emerging area of research has shown that there is a dual nature to these cytokines. Recent evidence has demonstrated that both type I and type II IFNs have immunoregulatory functions during infection and type II immune responses. In this review, we address the dual nature of type I and type II interferons and present evidence that both antiviral and immunomodulatory functions are critical during virus infection to not only limit virus replication and initiate an appropriate antiviral immune response, but to also negatively regulate this response to minimize tissue damage. Both the activating and negatively regulatory properties of type I and II IFNs work in concert with each other to create a balanced immune response that combats the infection while minimizing collateral damage.

## Introduction

Type I and II interferons (IFN) are cytokines produced during virus infection that are integral for regulating the immune response. Type I IFNs are well known for their ability to directly induce an antiviral response within infected and surrounding cells through the upregulation of molecules that can antagonize virus replication (1). As they are produced rather early on during an infection, type I IFNs are also essential for activating the antiviral innate immune response, such as natural killer (NK) cell effector functions (2). Type II IFN, known as IFN-γ, while sharing a similar nomenclature to type I IFN, signals through a different receptor and has effects that are independent from type I IFN. As a part of the innate immune response, they are predominantly produced by natural killer cells during infection (2). IFN-γ, like type I IFN, promotes antiviral immunity through its regulatory effects on the innate immune response and acts as a key link between the innate immune response and activation of the adaptive immune response (3). Beyond their antiviral effects, a growing amount of evidence suggests that type I and type II IFNs have immunoregulatory functions that are critical for dampening immunopathogenic mechanisms and minimizing collateral damage from the infection. Altogether, this review will build a framework and provide evidence demonstrating that these two cytokines are both critical for limiting virus replication and promoting a beneficial virus limiting response, while simultaneously dampening immunopathology. If we consider the world outside of virus infections, however, this fundamental duality of type I and II IFNs can be applied to numerous pathological processes, ranging from allergy to autoimmune diseases.

### Type I and II IFN production and signaling

Type I IFNs consist of a group of structurally similar cytokines and include 13–14 subtypes of IFN-α along with IFN-β, IFN-ε, IFN-κ, IFN-ω, IFN-δ, IFN-ζ, and IFN-τ (4, 5). As part of the innate immune antiviral response, these cytokines are rapidly produced after pattern-recognition receptor (PRR) stimulation (5). Current research suggests that an initial wave of IFN-β and IFN-α4 is produced and dependent upon IRF3 phosphorylation and NFκb activation (6–8). The initial type I IFN wave subsequently induces IRF7 phosphorylation and results in a positive feedback loop of increasing type I IFN release. Once produced, these cytokines all signal through the same receptor, the type I IFN receptor (IFNAR). IFNAR is composed of two subunits—IFNAR1 and IFNAR2—which when bound to type I IFN are endocytosed and activate their associated tyrosine kinases, Tyk2 and Jak1 (4, 9). The classical signaling cascade results in phosphorylation of STAT2 and STAT1, which forms a complex with IRF9, known as the IFN-stimulated gene factor 3 (ISGF3) (4). ISGF3 then leads to expression of IFN-stimulated genes (4). Beyond ISGF3, type I IFNs can also induce phosphorylation and dimerization of STAT3, STAT4, STAT5, and STAT6 and has been shown to induce activation of Rap1, CrkL, Map kinases, IRS-1 and -2, Vav, RAC1, and PI3-kinase signal transduction pathways (4, 10–14). Interestingly, IFN-β has been shown to additionally signal through the IFNAR1 subunit independent from IFNAR2 and carries through a non-canonical signaling pathway (15).

Type II IFN is predominantly produced by NK cells during the antiviral innate immune response (16). A multitude of evidence has shown that type I IFN, IL-12, IL-15, and IL-18 are all capable of inducing IFN-γ production from NK cells (17). NK cell IFN-γ is dependent upon STAT4 phosphorylation for its production. Once released, IFN-γ signals through the IFN-γ receptor (IFNGR), composed of IFNGR1 and IFNGR2 subunits. In the classical signaling pathway, ligation of IFN-γ to the IFNGR leads to activation of JAK1 and JAK2, resulting in homodimerization and phosphorylation of STAT1 (18). However, like type I IFN, IFN-γ has also been shown to signal through alternative pathways, including STAT4, Erk1/2, Pyk2, and CrkL, among others (18).

### Type I IFN: mastering the antiviral response

Type I IFN is one of the first cytokines produced during a virus infection. In the context of HSV-2 infection, for example, there is an initial wave of IFN-β production at 12 h post-infection, followed by both IFN-β and IFN-α production at 48 h post-infection (19, 20). This early production of type I IFN is critical for induction of both an antiviral response within infected and target cells, as well as activation of innate immune cells that will ultimately serve to control virus replication and activate the adaptive immune response to both clear the infection and generate memory to create a rapid response against future infections (21).

Type I IFN is a well-known stimulator of antiviral genes targeted against preventing virus replication from within target cells. When their production is stimulated by virus infection, type I IFN can act in an autocrine, paracrine, or systemic fashion. Their protective role during virus infection is highlighted by the increased mortality observed in mice deficient in the type I IFN receptor (*Ifnar*^−/−^) in comparison to their control counterparts when infected with a virus (22, 23). Upon ligation to its receptor, type I IFN has been shown to induce upwards of 300 ISGs. Of these 300 genes, 51 were found to contribute to host defense, while other genes contributed to inflammation, signaling, transcription, and immunomodulation, among other activities (24, 25). Further, De Veer et al. examined the ability of specific ISGs or combinations of ISGs to inhibit virus replication through overexpression of individual ISGs prior to virus infection (24). They found that many ISGs were capable of inhibiting virus replication, with some acting on a wide range of viruses, while others were only effective against particular viruses (24). Interestingly, they found that select ISGs enhanced virus replication in their experimental system (24). Antiviral ISGs can hinder virus replication through several mechanisms. Protein kinase R, for example, inhibits cellular translational functions (1). 2′5 OAS and RNaseL, on the other hand, degrade RNA (26, 27). Other ISG antiviral activities can prevent virion release, inhibit virus entry, and inhibit virus transcription (28).

Apart from their induction of antiviral ISGs, type I IFNs are key regulators of the innate immune response. Within the type I IFN literature, a theme has emerged wherein acute type I IFN production promotes beneficial antiviral responses, while chronic type I IFN production can have a suppressive and deleterious effect on the immune response. Within this section, we will examine the ability of type I IFN to promote antiviral functions in dendritic cells (DC), monocytes, and NK cells.

Dendritic cells are critical for activation of antiviral T-cells (29). Type I IFN stimulation has been shown to enhance MHC II expression and presentation of antigens as well as upregulate co-stimulatory molecules and promote DC maturation (29–32), Further evidence suggests that type I IFN is able to increase differentiation of plasmacytoid DCs into myeloid-derived DCs to increase T-cell activation (33).

Inflammatory monocytes are rapidly recruited to sites of infection, where they can then stimulate local and migrating immune cell antiviral function, promote inflammation, and differentiate into macrophages and DCs (34). At sites of inflammation, type I IFNs induce production of CCL2 to recruit inflammatory monocytes (2, 34). Type I IFN produced during vaginal HSV-1 infection induces tissue resident macrophages and DCs to produce CCL2 to recruit and initial population of inflammatory monocytes, which then enact a positive feedback loops to produce more CCL2 to attract further inflammatory monocytes (35). A similar phenomenon has been observed during vaginal HSV-2 infection, influenza infection, and inflammatory monocyte recruitment to the brain during LPS-induced systemic inflammation (2, 36, 37), With influenza infection, absence of IFNAR resulted in differentiation of Ly6C intermediate expressing monocytes rather than Ly6C^hi^ inflammatory monocytes, which additionally had a different phenotype (36). Further, Seo et al. demonstrated that *Ifnar*^−/−^ bone marrow had a significantly decreased differentiation of hematopoietic cells into inflammatory monocytes in the presence of influenza infection (38). In regards to macrophages, Type I IFN has more of a suppressive function and will be discussed below.

Type I IFN and antiviral NK cell functionality are tightly interwoven, where type I IFN has emerged as a key NK cell regulator. Like their monocyte counterparts, type I IFN has been implicated in NK cell recruitment to sites of inflammation. During a vaginal HSV-1 infection, type I IFN was required to induce epithelium production of CCL3, CCL4, and CCL5 to recruit NK cells to the vaginal mucosa (35). Further, type I IFN has been implicated in the activation of NK cell antiviral functions. During an infection, NK cells have several weapons under their belt that they can utilize to combat infection. When activated, they can release IFN-γ, cytotoxic granules, and induce cell death of infected cells. Type I IFN has been implicated in both NK cell cytotoxicity and NK cell IFN-γ production. Mice deficient in STAT1, a key transcription factor downstream of type I IFN receptor, have been shown to have decreased NK cell cytotoxicity and increased virus-induced mortality in comparison to control mice (39). In the context of NK cell IFN-γ production, type I IFN is essential for this process in multiple virus infections, including MCMV, adenovirus, vaccinia virus, and HSV (2, 40–43). Type I IFN has been shown to act directly on NK cells to induce their release of IFN-γ in the context of adenovirus, vaccinia virus, and LCMV infections, whereas other evidence suggests that type I IFN stimulates DCs to trans-present IL-15 to activate NK cells in MCMV infection (2, 40–44). Recently, we have provided evidence demonstrating that NK cell IFN-γ production relies on type I IFN induction of IL-18 from inflammatory monocytes, rather than DCs in a mucosal HSV-2 infection (2). Our differing results may stem from the route of infection, where previous evidence used *in vitro* systems or non-mucosal routes of infection.

### Type I IFN negative regulation: beyond interfering with infection

As more evidence emerges, there is a greater understanding and appreciation for the suppressive and negative regulatory aspects of type I IFN. Early on, studies had shown that type I IFN exerted anti-proliferative effects on immune cells and cell lines (45, 46). Recently, Thomas et al. elegantly demonstrated that while all type I IFN subtypes were capable of inducing an intracellular antiviral response, the affinity of an individual type I IFN subtype to the type I IFN receptor largely determined the ability of type I IFN to inhibit cellular proliferation (47). The antiproliferation effects of type I IFN required higher binding affinities to IFNAR (47). Beyond proliferation, type I IFN can suppress innate immune cell functions as well.

While an acute infection and upregulation of type I IFN is beneficial for enhancing DC activation of T-cell adaptive functions, a chronic infection with sustained type I IFN production has been shown to dampen DC expansion and induce a suppressive phenotype. In chronic LCMV infection, a persistent type I IFN signature prevented BM differentiation and proliferation of conventional DCs (48, 49). Further, stimulation of splenic DCs with IFN-β, *in vivo*, resulted in a decrease in total CD11c+ cell number. In addition to reducing DC expansion, a chronic type I IFN signature was shown to upregulate PD-L1 expression and IL-10 in both DCs and macrophages (50, 51).

Type I IFN largely has a suppressive effect on macrophages. The literature largely suggests that it downregulates their expression of the IFN-γ receptor, making them less sensitive to IFN-γ stimulation (52). In certain bacterial infections, such as *francisella tularensis* and *mycobacterium tuberculosis*, type I IFN signaling is detrimental to the host (53–56). The ability of type I IFN to downregulate the IFN-γ receptor on macrophages likely contributes to this phenomenon.

As mentioned previously, type I IFN has been shown to be critical for inducing the antiviral functions of NK cells. Conversely, and almost paradoxically, type I IFN has also been shown to suppress the very functions that it enables. During LCMV infection, Teijaro et al. found that blocking the type I IFN receptor rescued IFN-γ production from NK cells (48). Further, persistent type I IFN production can induce expression of PD-L1 ligands, which is a mechanism that can suppress NK cell antiviral function (48). Though administration of pegylated IFN-α2 therapy resulted in an increased NK cell activation, TRAIL, and CD107a receptor expression in HCV-infected individuals, there was a concomitant reduction in IFN-γ+ NK cells within the PBMC compartment (57, 58). This contradictory effect of type I IFN may stem from the timing and magnitude of type I IFN produced or a shift in transcription factor association with the type I IFN receptor. In a *listeria monocytogenes* infection, exogenous IFN-β administered at an earlier time point during infection was able to activate NK cells and promote clearance of the infection, whereas the endogenous IFN-β produced at 24 h post-infection resulted in an impaired NK cell response (59). Further, Marshall et al. found that stimulation of NK cells with supernatants from CpG-stimulated pDCs in addition to IFN-α suppressed IFN-γ release from NK cells (60). In a seminal study from Miyagi et al. they demonstrated that stimulation of NK cells with type I IFN shifted the balance of transcription factors from a STAT4 association with the type I IFN receptor, which upon phosphorylation and nuclear translocation resulted in an initial burst of IFN-γ, to a STAT1 association that subsequently led to inhibition of NK cell IFN-γ production (61). Thus, as increasing amounts of type I IFNs are released during infection, this leads to an increasing shift in association between STAT1 and IFNAR and ultimately inhibition of IFN-γ production from NK cells.

Along with promoting antiviral functions (and later limiting these very same functions), type I IFN has been shown to limit damaging immune responses that can lead to tissue pathology and collateral damage. In a model of influenza infection, absence of the type I IFN receptor resulted in significant virus-induced immunopathology. Duerr et al. demonstrated that this pathology was mediated by an upregulation of type 2 cytokines from unregulated innate lymphoid type 2 cells (ILC2s) (62). Thus, type I IFN suppresses ILC2 function during virus infection. Type I IFN was also found to suppress pro-inflammatory NOS2+ Ly6C^lo^ monocyte function (36). Moreover, type I IFN dampens recruitment of neutrophils by suppressing epithelial CXCL1 and CXCL2 production during virus infection (35, 38, 63). Not only can neutrophils produce a multitude of molecules and proteases that can promote inflammation and tissue damage, they have been shown to instigate rhinovirus-induced asthma exacerbations in mice (64, 65). A table comparing the effects of type I IFN on the innate immune response is summarized in Table 1.

**Table 1:** The role of type I IFN in regulating the antiviral innate immune response.

**Cell type**	**Positive Regulation**	**Negative Regulation**
**DCs**	T-cell activation: –Increases surface expression of CD40, CD80, CD86, OX40L, and MHC II (29, 31) –Stimulation of terminal DCs enhances MHC II and B-7 expression (32) –Sustains Ag processing and MHC II expression (30) Suppressive functions: –Chronic type I IFN stimulation increases expression of IL-10 and PD-L1 (50, 51) Differentiation: –pDC conversion into mDC (33)	Differentiation/proliferation: –Chronic type I IFN stimulation reduces BM-derived cDC differentiation and proliferation (48, 49) –Stimulation during the differentiation process inhibits CD11c, MHC-II, and B-7 expression (32)
**Inflammatory monocytes**	Recruitment: –Induction of CCL2 for inflammatory monocyte recruitment (2, 34, 36, 37) Differentiation: –Absence of IFNAR leads to decreased Ly6C^hi^ inflammatory monocyte differentiation and results in increased levels of Ly6C^intermediate^ monocytes (36)	Function: –Downregulation of IFNγR expression and subsequently NOS2 expression (36)
**Macrophages**	Function: –Upregulation of IL-10 and PD-L1 (50, 51)	Function: –Downregulation of IFNγR expression (52)
**Neutrophils**	No evidence of activation	Recruitment: –Suppresses CXCL1 and CXCL2 production (35, 38, 63)
**NK cells**	Recruitment: –Induction of CCL3, CCL4, CCL5 for NK cell recruitment (35) Activation: –Implicated in STAT-1-mediated cytotoxicity (39) –Required for IFN-γ production (2, 40, 41, 42, 43)	Suppression of IFN-γ due to: –Chronic type I IFN (48, 57, 58) –Increased levels of type I IFN (60) –Timing of type I IFN—early release results in activation, late results in inhibition (59)
**ILC2**	No evidence of activation	Proliferation: –Reduces ILC2 proliferation (62) Function: –Reduces expression of IL-5, IL-6, and IL-13 (62)

### Unweaving the dual nature of type I IFNs

Within the literature, various themes are emerging that provide an explanation for this underlying dual functionality of type I IFN. First, acute virus infections and transient type I IFN production appears to promote antiviral responses from innate immune cells, while chronic infections with persistent type I IFN signatures result in a dampened antiviral response (66). This is particularly evident in the cases of chronic LCMV, which led to deterioration of the lymphoid architecture and T-cell suppression mediated by increased PD-L1 expression on DCs (48, 49). In simian immunodeficiency virus (SIV) infection, early administration of type I IFN resulted in a reduction in viral load, while chronic administration of type I IFN resulted in an increased level of virus and CD4+ T-cell depletion (67, 68). Second, the timing and magnitude of type I IFN produced can result in differing type I IFN responses, as previously discussed.

A growing body of evidence has revealed that individual subtypes of type I IFN can have differing effects, despite signaling through the same receptor. Indeed, stimulation of DCs with different subtypes of type I IFN resulted in varying profiles of receptor expression and cytokine production (69). Additionally, pre-treatment of influenza-infected mice with the same dose of different type I IFN subtypes resulted in varying levels of virus replication, with IFN-α5 and IFN-α6 having the greatest reduction in viral load (70). Their differing affinities for the type I IFN receptor, length of receptor binding, level of type I IFN receptor expression, and innate cellular differences may underlie the ability of these type I IFN subtypes to induce different responses (71). This is outlined in greater and more elegant detail in a review by Gideon Schreiber (71). In the context of virus infection, however, we hypothesize that type I IFN acts to optimize the antiviral response by both activating and enhancing beneficial innate immune cell function, while limiting detrimental and pathological immune responses that can cause unnecessary tissue damage.

### Type II IFN: an antiviral state of mind

IFN-γ is an important component of the innate antiviral response and is predominantly produced by NK cells or innate lymphoid type 1 cells (2, 72, 73). In the context of HSV-2 infection, absence of IFN-γ production results in increased virus replication and decreased survival (74, 75). Indeed, IFN-γ has been shown to induce NO production, a potent inhibitor of virus replication, from surrounding cells (72, 76). As well, IFN-γ can induce intracellular antiviral programs, including PKR, as a resultant overlap in their gene expression with type I IFNs (77). Beyond that, however, IFN-γ itself has been demonstrated to impact the function of the surrounding innate immune cells, including macrophages and DCs.

The impact of antiviral IFN-γ on antigen presenting cells (APCs) is to enhance stimulation of the adaptive antiviral response to both clear the infection and generate memory as a safe-guard for future infections (78, 79). Thus, it is a critical propellant of the Th1 response. IFN-γ stimulation enhances the antigen presentation process during T-cell priming. It has been shown to increase various aspects of antigen presentation, including efficiency, quantity, quality, and diversity of peptides being loaded into the MHC I receptor (80). Along with MHC I, IFN-γ increases MHC II expression and maturation of DCs (81). Further, it induces the expression of IL-12 and co-stimulatory CD80 in antigen-presenting cells, which is a critical component of Th1 polarization (82–84).

With respect to macrophages, IFN-γ induced NO production from these cells not only inhibits virus replication, but also potently vasodilates blood vessels to decrease blood flow and allow for increased extravasation of recruited immune cells to the site of infection and inflammation (80). Further, IFN-γ has been shown to “prime” macrophages to release reactive oxygen species, through the upregulation of cellular components required for this function (85). IFN-γ also appears to increase macrophage receptor-mediated phagocytosis through the upregulation of complement receptors, though this has been observed more so in bacterial infections, rather than viral (86). Further, IFN-γ promotes polarization of macrophages to an M1 phenotype and primes these cells to produce pro-inflammatory cytokines IL-12, TNF-α, and IL-1β (87, 88).

### Type II IFN negative regulation: an emerging role

IFN-γ has many overlapping features with type I IFNs, including suppression of type 2 immune responses and inhibition of proliferation. In the context of virus infection, however, we believe that IFN-γ released during the innate immune response has more of a supportive role in this respect as it is less potent in its effects in comparison to type I IFNs. Aside from type I IFN, IFN-γ has a number of immunoregulatory functions that serve to optimize the antiviral response and limit overzealous responses that could lead to collateral damage.

An optimal antiviral response involves both activating beneficial immune responses, while simultaneously inhibiting impractical and potentially damaging responses. In the context of virus infection, IFN-γ is a prototypical Th1 promoting cytokine. Further, evidence from Kang et al. demonstrates that IFN-γ plays a critical role in not only polarizing macrophages to an M1 phenotype, but actively suppresses the M2 polarization pathway (87). However, recent evidence has revealed that type I IFN is capable of suppressing type 2 immunity. Independently, both Duerr et al. and Moro et al. demonstrated that, similar to type I IFN, IFN-γ is able to suppress ILC2 proliferation and type 2 cytokine production (62, 89). Indeed, *in vivo* administration of IFN-γ potently suppressed IL-33-induced ILC2 proliferation, which was dependent upon STAT1 signaling (62). In the context of RSV infection, *Stat1*^−/−^ mice, a transcription factor downstream of both type I and type II IFNs, led to increased lung pathology because of increased cytokine production from ILC2s and ILC3s (90). Further, in a mouse model of influenza infection, administration of IFN-γ suppressed ILC2 function while deficiency of IFN-γ led to increased IL-5 and amphiregulin release from ILC2s. These authors ultimately found that the suppressive effects of IFN-γ on ILC2 function led to increased lung pathology (91).

Along with dampening immune responses, there is evidence demonstrating that IFN-γ can indirectly induce immunoregulatory effects through the upregulation of PD-L1 and differentiation of myeloid derived suppressive cells. In conjunction with GM-CSF, IFN-γ was shown to differentiate monocytes into myeloid derived suppressor cells (MDSCs) *in vitro* (92). In a mouse model of endotoxemia, IFN-γ has also been shown to upregulate PD-L1 on neutrophils (93). A table comparing the effects of IFN-γ on the innate immune response is summarized in Table 2.

**Table 2:** The role of IFN-γ in regulating the antiviral innate immune response.

**Cell type**	**Positive Regulation**	**Negative Regulation**
**APCs**	T-cell activation: –Promotes DC maturation (81) –Increases MHC I and MHC II expression (80, 81) –Enhances efficiency, quantity, quality, and diversity of MHC I Ag-loading (30) –Increases expression of IL-2 and CD80 (82, 83, 84)	No evidence of negative regulation
**Macrophages**	Function: –Induces NO production (80) –Primes macrophages for ROS release (85) –Increases phagocytosis (86) –Polarization to M1 phenotype (87, 88)	No evidence of negative regulation
**Neutrophils**	Function: –Increases PD-L1 expression (93)	No evidence of negative regulation
**MDSC**	Function: –Upregulation of PD-L1 (92) Differentiation: –Enhances differentiation of MDSCs (92)	No evidence of negative regulation
**ILC2**	No evidence of activation	Proliferation: –Reduced ILC2 proliferation (62, 89) Function: –Reduced expression of IL-5, IL-6, and IL-13 (62, 89) –Reduced expression of amphiregulin (89)

### Understanding the dual nature of IFN-γ: unraveling the paradox

Similar to type I IFNs, IFN-γ has both seemingly paradoxical activating and suppressive functions on the innate antiviral response. These functions can be teased apart if we examine the cell type that IFN-γ is acting upon and bring other cytokines into the picture with IFN-γ. If we consider macrophages, IFN-γ has complementary effects on inducing an antiviral macrophage function. IFN-γ induces NO production, enhances macrophage antigen presenting function, and an overall M1 phenotype while actively suppressing the M2 phenotype (72, 80, 87). Similar to macrophages, IFN-γ predominantly increases antigen presentation function of DCs. Further, IFN-γ has a predominantly suppressive effect on ILC2 cells (62).

IFN-γ as a cytokine rarely acts alone and its effects should be considered in conjunction with other cytokines present in the local microenvironment. The combinatorial effect between IFN-γ and other cytokines likely plays a role in the ultimate outcome of IFN-γ stimulation. Indeed, both IFN-γ and TNF-α have been shown to synergize in the upregulation of iNOS in macrophages. Salim et al. used mathematical modeling to dissect out the roles of each cytokine and found that TNF-α was largely responsible for the timing of iNOS induction by inducing a rapid response, whereas IFN-γ impacted the levels and concentrations of NO production (94). Further, the role of IFN-γ in the *in vitro* differentiation process of MDSCs required an initial priming with GM-CSF. Ribechini et al. found that GM-CSF altered the signaling pathway of IFN-γ allowing it to differentiate monocytes into MDSCs (92). In a recent article by Zha et al. they found that IFN-γ was able to suppress the functions of gp130 cytokines, particularly the ability of OSM, to differentiate mesenchymal stem cells through the upregulation of STAT1, concomitant decrease in STAT3 activation, and internalization of the gp130 receptor (95). Thus, IFN-γ can both be altered by additional cytokine signaling as well as regulate the signaling pathways of other cytokines.

### Putting the pieces of the puzzle together

**Figure 1:**
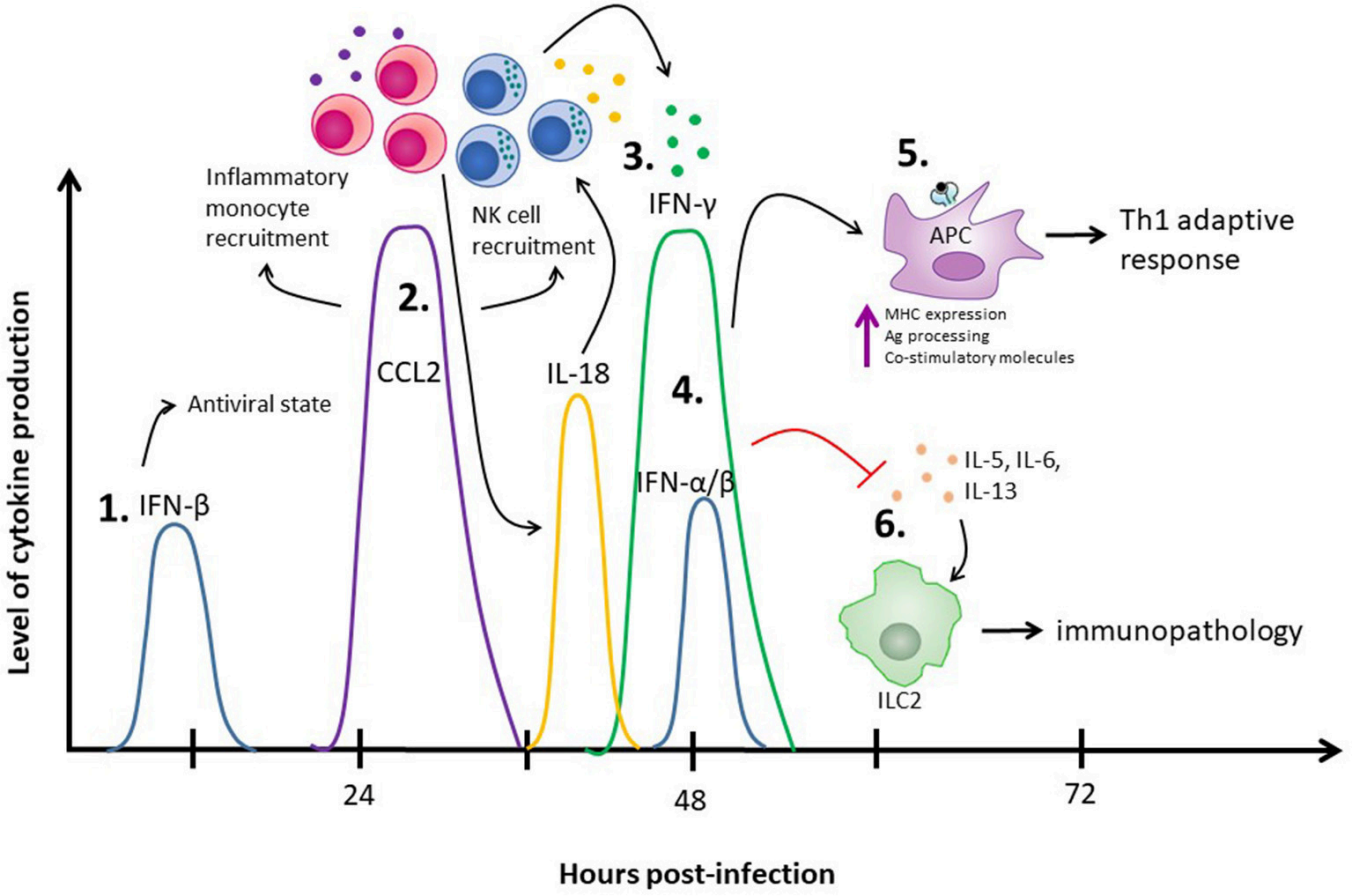
The role of IFNs in the innate immune response to HSV-2 infection. (1) IFN-β is produced at 12 h post-infection and through autocrine and paracrine signaling places surrounding cells into an antiviral state. (2) The IFN-β produced at 12 h post-infection also increases production of CCL2 between days 1 and 2 post-infection, which results in inflammatory monocyte recruitment and has been implicated in NK cell recruitment. (3) The recruited inflammatory monocytes result in release of IL-18, which stimulates NK cells to produce IFN-γ at 48 h post-infection. (4) A second wave of type I IFNs, including both IFN-α and IFN-β, are detected at 48 h post-infection. (5) Both IFN-γ and the type I IFNs produced at 48 h post-infection enhance APC antigen presentation capacities to stimulate a Th1 adaptive immune response. (6) Simultaneously, the type I IFNs at 48 h inhibit ILC2-mediated virus-induced immunopathology. IFN-γ, supporting the negative regulatory effects of type I IFN, also suppresses ILC2-mediated immunopathology.

As we start to put the pieces of this type I and type II IFN puzzle together, we can see that these two cytokines act in concert with one another to limit virus replication and encourage an antiviral adaptive immune response while suppressing detrimental functions of other immune cells to limit tissue pathology. Using vaginal HSV-2 infection as an example, we find that there are multiple waves of type I IFN production, starting with IFN-β at 12 h post-infection (20). This early wave of IFN-β is likely responsible for the induction of MCP-1-mediated inflammatory monocyte recruitment, ultimately leading to IL-18-induced NK cell IFN-γ production (2). From there, we've observed a second wave of type I IFN production, both IFN-α and IFN-β, at 48 h post-infection (19). Along with type I IFN, there's a sharp increase in IFN-γ from NK cells at 48 h post-infection (16). The IFN-γ released from NK cells is also negatively regulated by type I IFN, as NK cells lacking IFNAR have increased IFN-γ production in the context of HSV-2 infection (2). This second wave of type I and II IFNs likely work in concert with each other to promote APC maturation, upregulation of co-stimulatory molecules, and antigen processing and presentation to promote Th1 polarization, while simultaneously suppressing ILC2-mediated immunopathology (Figure 1).

Without type I IFN, and potentially type II IFN, there is uncontrolled virus replication coupled with uncontrolled inflammation that work together to cause tissue demise. On the other hand, a chronic type I IFN signature is detrimental as it can result in immunosuppression and increased virus replication. Thus, we believe a *balanced and appropriate* type I IFN response is required to regulate an optimal and advantageous antiviral innate immune response.

### Clinical implications: going beyond infection

While the focus of this review has been on type I and II IFNs and their ability to control the innate immune response, IFNs have been implicated in several non-infectious pathological conditions. Select autoimmune diseases, the most prominent being systemic erythematous lupus (SLE), have high type I IFN signatures associated with their pathology (96). An antibody targeting human IFNAR has recently been developed to block this signature with therapeutic benefit (97). On the other hand, IFN-β therapy has had success in treating multiple sclerosis (98). Indeed, the concepts discussed in this review are relevant in the context of pharmacotherapies targeting the type I and type II IFN pathways. This begs the question: what is the role of type I IFN outside of virus infection? A growing amount of evidence has shown that type I IFN production is not isolated to infectious disease stimuli, it can be produced during any inflammatory insult. Thus, our fundamental understanding of the innate immune response during virus infection has an underlying application to many disease processes, beyond virus infection.

## Author contributions

AL generated themes and ideas and wrote the manuscript. AA guided and edited the manuscript.

### Conflict of interest statement

The authors declare that the research was conducted in the absence of any commercial or financial relationships that could be construed as a potential conflict of interest.
